# Mechanisms of Plant Epigenetic Regulation in Response to Plant Stress: Recent Discoveries and Implications

**DOI:** 10.3390/plants13020163

**Published:** 2024-01-07

**Authors:** Mukhtar Iderawumi Abdulraheem, Yani Xiong, Abiodun Yusuff Moshood, Gregorio Cadenas-Pliego, Hao Zhang, Jiandong Hu

**Affiliations:** 1 Department of Electrical Engineering, Henan Agricultural University, Zhengzhou 450002, China or m.iderawumi@gmail.com (M.I.A.); xiongyani888@163.com (Y.X.); yusufbinmasud01@stu.henau.edu.cn (A.Y.M.); hao.zhang@henau.edu.cn (H.Z.); 2 Henan International Joint Laboratory of Laser Technology in Agriculture Science, Zhengzhou 450002, China; 3 State Key Laboratory of Wheat and Maize Crop Science, Zhengzhou 450002, China; 4 Centro de Investigación en Química Aplicada, Blvd. Enrique Reyna 140, Saltillo 25294, Mexico; gregorio.cadenas@ciqa.edu.mx

**Keywords:** plant stress, epigenetic regulation, DNA methylation (DM), genome-wide profiling, histone modification (HM), non-coding RNA, chromatin remodeling complex

## Abstract

Plant stress is a significant challenge that affects the development, growth, and productivity of plants and causes an adverse environmental condition that disrupts normal physiological processes and hampers plant survival. Epigenetic regulation is a crucial mechanism for plants to respond and adapt to stress. Several studies have investigated the role of DNA methylation (DM), non-coding RNAs, and histone modifications in plant stress responses. However, there are various limitations or challenges in translating the research findings into practical applications. Hence, this review delves into the recent recovery, implications, and applications of epigenetic regulation in response to plant stress. To better understand plant epigenetic regulation under stress, we reviewed recent studies published in the last 5–10 years that made significant contributions, and we analyzed the novel techniques and technologies that have advanced the field, such as next-generation sequencing and genome-wide profiling of epigenetic modifications. We emphasized the breakthrough findings that have uncovered specific genes or pathways and the potential implications of understanding plant epigenetic regulation in response to stress for agriculture, crop improvement, and environmental sustainability. Finally, we concluded that plant epigenetic regulation in response to stress holds immense significance in agriculture, and understanding its mechanisms in stress tolerance can revolutionize crop breeding and genetic engineering strategies, leading to the evolution of stress-tolerant crops and ensuring sustainable food production in the face of climate change and other environmental challenges. Future research in this field will continue to unveil the intricacies of epigenetic regulation and its potential applications in crop improvement.

## 1. Introduction

Plants are fascinating organisms, with the remarkable ability to modulate their developmental processes and adjust to their surroundings through epigenetic modifications. These modifications extend beyond the realm of genetically encoded factors, adding an extra layer of regulation [1,2]. In the plant kingdom, epigenetic inheritance takes two forms: transferring information not encoded in DNA between generations and preserving epigenetic modifications within an individual reset between generations [3,4]. Comprehending the intricacies and operations that differentiate the two forms of epigenetic inheritance holds great significance. This area of research is particularly fascinating because stress can stimulate stress-signaling pathways which enhance stress gene responses; this knowledge can also be used to develop strategies for improving crop yield, quality, and stress resistance, which are benefiting agriculture and the ecosystem [5,6]. Plants use epigenetic regulation to enhance immunity and variation during pathogen and pest interactions [7,8,9,10,11]. Recent studies of epigenetic mechanisms significantly impact how plants react to non-living factors and well-understood signal transduction mechanisms [5,12].

It should be noted that inherited stress tolerance mechanisms vary among plant species based on genetic makeup, intensity, and stress duration [13]. Mutations in DNA sequences cause trait variations, which plant breeders use to improve plant populations due to alterations in chromatin states [14]. Notably, plants can remember and learn from their experiences, making them highly adaptable to their environments [15,16]. During stress responses, epigenetic modifications are known to significantly ensure the reprogramming and gene expression of the plant’s transcriptome. These changes are mediated by modifications to the chromatin structure, like “DM, histone modifications (HM), and non-coding RNA molecules”. Instead, it involves modifications to the structure of DNA or associated proteins that can influence gene activity [17]. These modifications can be stable and passed on to subsequent generations, allowing plants to transmit stress memories across generations [14].

Recent research has uncovered that identical plants can exhibit DM changes when subjected to varying stressors [2,18,19,20]. Remarkably, apomictic *Taraxacum officinale* plants exposed to abiotic stress displayed notable differences in DM, regardless of the specific type of stress. These indicate that epigenetic inheritance may be a pivotal factor in plant adaptation, even when genetic diversity among individuals is absent. Stress-induced methylation patterns are influenced by stress type, genotype, tissue, and organism, which affect stress-responsive gene regulation [21,22]. In plants, DM occurs predominantly at cytosine residues in a CG context (CG methylation), but it can also occur in other sequence contexts, such as CHG and CHH (where H is A, C, or T) [23]. DMs and HMs alter gene expression by inhibiting transcription factor binding or modifying DNA accessibility to regulatory proteins [24].

Epigenetic regulations are vital for plant processes such as growth, development, reproduction, and pathogen resistance, as well as improving adaptability to environmental stressors like temperature, salinity, and nutrient scarcity [12,25,26,27,28]. The manipulation of epigenetic processes requires tremendous effort to enhance crop yield, growth, quality, and productivity. These, in turn, contribute to sustainable agriculture, where epigenetic mechanisms regulate critical agronomic traits in crops via DM, histone modifications, and small RNAs that affect gene expression and impact growth, seeding, germination, and fruit development [29]. The impact of these epigenetic mechanisms is felt directly in crop productivity, yields, and quality.

**Figure 1:**
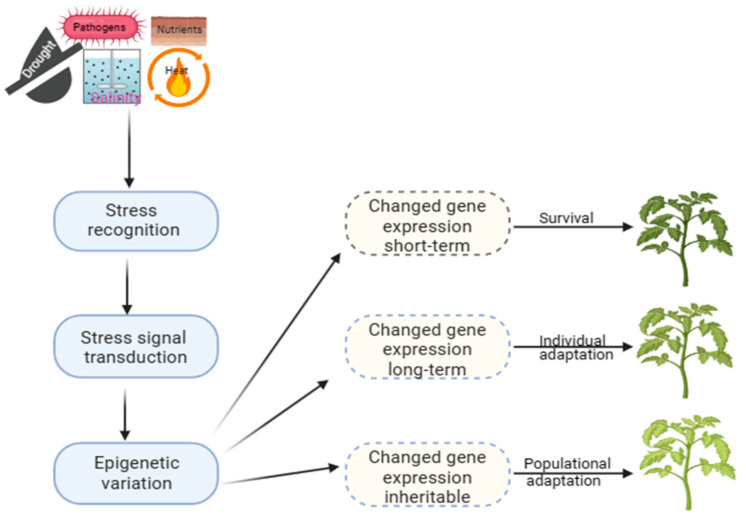
Epigenetic processes and mechanisms of plant adaptation to stress.

Long-term modifications play a significant role in evolution, providing a stable molecular basis for phenotypic plasticity. At the same time, short-term mechanisms, on the other hand, are crucial for surviving under stress (Figure 1). This adaptation allows the selection of offspring better suited to a constantly changing environment, which can be observed in natural populations with similar genetic makeup, indicating that it is an epigenetic trait [12]. Developing strategies to improve crop productivity under challenging environmental conditions requires a comprehensive understanding of plant epigenetics and stress responses. However, there is still a lack of direct evidence linking epigenetic changes to phenotypic plasticity in plants exposed to varied environments or different types of stress [30]. Recent research has shown that plants can regulate gene expression through DM patterns, which can be altered dynamically under stress to adapt and thrive under harsh conditions [12,31,32].

Research has demonstrated that certain enzymes are implicated in stress-induced epigenetic changes, like DRM2, a DNA methyltransferase, which plays a critical role in stress-triggered alterations of DM [19,33,34]. Stress-responsive HMs have also been linked to histone acetyltransferases and histone deacetylases. To develop plants that can minimize the effects of stress, it is necessary to examine the mechanisms involved in detail. This review discusses and summarizes the recent discoveries of mechanisms related to plant epigenetic regulation in response to plant stress and provides insights into how plants adapt and survive under challenging conditions and their generational heritability.

## 2. Mechanisms of Plant Epigenetic Regulation

**Figure 2:**
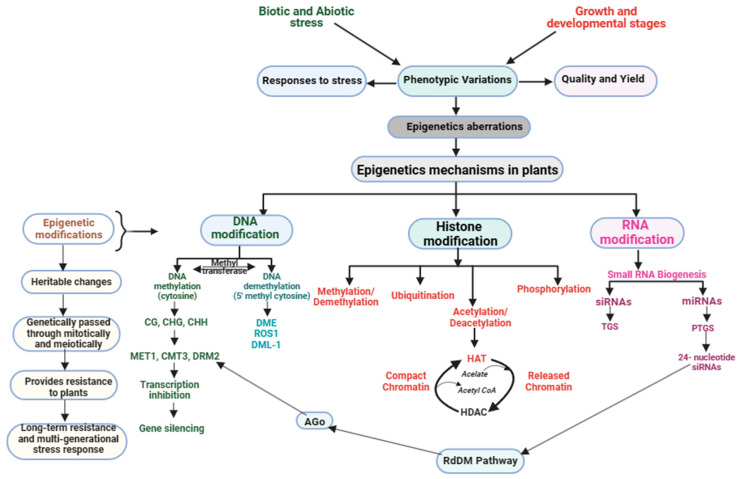
Plant epigenetic modifications in response to stress management during growth and development.

In response to stress, plants employ a range of epigenetic mechanisms to fine-tune gene expression. These tactics comprise DM, histone modifications, small RNA-mediated gene silencing, and chromatin remodeling (Figure 2). Regulating gene expression and maintaining genome stability are crucial functions, with each approach playing a distinct and essential role.

### 2.1. DNA Methylation (DM) 

Epigenetic modification through DM is a well-researched process in the plant kingdom. This process involves adding a methyl group to DNA’s cytosine residues, specifically at CpG dinucleotides. Stress-induced changes in DM patterns can significantly impact gene expression and phenotype plasticity [35]. Recent studies have shown that DM is a dynamic process that responds to various environmental stresses [23,32,34]. It participates in preventing certain transcription factors from binding to DNA and attracting chromatin-modifying proteins [36]. This process also determines histone modification patterns and helps recruit repressor complexes that contain HD7ACs, DNMTs, and MBD proteins. DM, unlike DNA sequence alterations, will result in complex DM states in crossbreeding populations, but it still has the potential to create novel and desirable phenotypes that genetic variety cannot provide [37,38]. It was further confirmed that, because DM is linked to gene expression, alterations in the methylation of areas that influence gene expression, such as cis-elements, may result in new gene expression and a new phenotype [37].

Plants can react to stress by adding a methyl group to DNA through DM. In *Arabidopsis thaliana*, for example, a gene called ATDM1 is responsible for drought stress response by methylating specific genes responsible for drought tolerance [39]. Pathogen infection can lead to DM changes, activating or repressing genes for defense. DM can silence genes and possibly recruit proteins that modify histones, leading to a more condensed chromatin structure and further repression of gene expression [24].

#### 2.1.1. Mechanisms of DNA Methylation in Plant Development

DM is a complex process that involves multiple enzymes and cofactors. The process starts with the recognition of a CpG dinucleotide by a DNA methyltransferase enzyme [17]. This modification alters the chromatin structure, leading to gene transcription suppression due to the ability of DM to regulate gene expression during plant development and stress response. It regulates important plant traits such as leaf structure, disease resistance, and environmental stress resistance [31,40]. In *Arabidopsis thaliana*, DM effectively suppresses the expression of specific genes involved in flower development, thereby causing a delay in the flowering process [39]. This suppression is achieved through the methylation of distinct CpG islands in these genes’ promoter regions (Figure 2). In addition to regulating flowering, DM also plays a part in controlling leaf morphology, where it represses the expression of genes responsible for shaping and sizing leaves, resulting in the formation of smaller leaves [41]. Research has found that *P. syringae* pv. tomato (Pst), a type of bacterial pathogen, can trigger defense and hormone pathways via DM [17,42]. *Arabidopsis* uses a mechanism to enhance its resistance to the pathogen and prevent downy mildew disease caused by *Hyaloperonospora arabidopsidis* (Hpa). Additionally, DNA hypermethylation plays a crucial role in improving the plant’s immunity to two fungal pathogens, *Plectosphaerella cucumerina*, and *Alternaria brassicicola*, in *Arabidopsis* [43].

#### 2.1.2. Role of DNA Methylation in Plant Stress Response

DM is essential for regulating gene expression and ensuring plant genome stability. Adding a methyl group to the cytosine residue of DNA creates 5-methylcytosine (5 mC) [4,39]. This 5 mC is involved in various biological processes (Figure 2), such as genome stability, transcriptional inactivity, developmental regulation, and response to environmental stress [36,44]. It acts as a repressive marker that suppresses gene expression, and its levels are regulated by both methylation and demethylation reactions [4]. DM can occur by either active or passive means, and its manipulation patterns could enhance crop yield, disease resistance, and tolerance to environmental stresses [40,45]. A study on salt-tolerance rice varieties and salt-sensitive has revealed that variations in global DM levels play a significant role in response to salt stress in regulating gene expression [46]. The research found that, under high salinity stress, promoter and gene body methylation levels are critical in regulating gene expression in a genotype and organ-specific manner. 

Furthermore, the study also showed that plants responded to high salinity by reducing DM levels, which is associated with the upregulation of the DNA demethylase (DRM2) gene. Interestingly, this upregulation was observed only in the salt-sensitive cultivar and not in the salt-tolerant cultivar. These findings suggest that differential DM patterns can impact salt stress tolerance in plants. Another study on rice cultivars under salt stress found significant changes in roots with minor changes in leaves [47]. The results suggest demethylation, with some persistent changes even after stress removal. The difference may be due to different detection methods or different rice lines used. An apple study underlined the significance of epigenetic modifications in response to dormancy produced by low temperatures. High freezing temperatures reduced total methylation, which resulted in the resumption of active development and subsequent fruit set in apples [48,49]. Research in *Populus trichocarpa* demonstrated that drought stress treatment might modify DM levels, altering the expression patterns of numerous drought stress-responsive genes [50], although the molecular mechanism behind this induction is unknown. The network and various plant species involved in epigenetic modifications in response to abiotic stress are shown in Table 1. The findings from the studies are itemized in the table as changes in various DMs under stress and shed light on plant responses to adverse conditions.

Wang et al. [41] demonstrated that drought stress induces changes in DM patterns in various plant species’ stress responses. In *Arabidopsis thaliana*, drought stress leads to global DNA *hypomethylation*, particularly in repetitive sequences and transposable elements [68]. This *hypomethylation* is associated with the upregulation and enhancement of drought-tolerance plant and responsiveness genes [44]. Within laboratory settings, specific stress treatments, such as extended or repeated exposure to elevated temperatures, may activate transgenes or TEs and impact surrounding genes [45]. Conversely, research has shown that *Oryza sativa* transcriptional regulation may cause transient hypermethylation of TEs near stress-inducing genes in low-phosphate responses [69]. Moreover, certain DNA demethylases have demonstrated the capacity to focus transcriptional regulation techniques available for enhancing stressful genes [70].

It is worth noting that environmental stressors cause DM and demethylation in various plant species [54,71]. Even in the absence of the original stress, these alterations can be retained and passed down to the progeny/offspring. However, the shift in DM is not consistent between stress events and plant species. For example, Eichten et al. [72], demonstrated that DM patterns in maize are inconsistent when the plants are exposed to a cold, heat, and UV irradiation. As a result, the inheritance of alterations in DM in corn, presumably connected to phenotypic changes, is unlikely to be strong. However, DM has demonstrated some consistency in terms of heredity in other tests. In rice, for example, a methyl-sensitive amplified fragment polymorphism study indicated that laser-induced DM is heritable and has triggered the production of micro-inverted-repeat transposable elements (MITEs) [73].

Similarly, Pathak et al. [74] confirmed that in rice (*Oryza sativa*), salt stress leads to both hypermethylation and hypomethylation of specific genomic regions. Research has shown that changes in DM can alter gene expression patterns and increase salt tolerance [35]. In addition, pathogen infection can cause changes in DM patterns in plants. For example, infection with *Pseudomonas syringae*, a bacterial pathogen, can lead to dynamic changes in DM patterns, particularly in defense-related genes in *Arabidopsis*. These changes activate defense responses and improve resistance to pathogen infection [32,43]. In *Zea mays*, DM patterns in genes involved in defense responses change due to insect herbivory, resulting in improved resistance to herbivory [75,76]. These DM modifications are linked to altered gene expression patterns [77]. More research is needed to comprehend how DM regulates stress-responsive gene expression and how these epigenetic modifications are inherited across generations.

### 2.2. Histone Modification (HM)

Chromatin and gene expression are regulated by histone modifications (HMs), which can either activate or repress gene expression (Figure 2), depending on the type of HM and its location [78]. Research shows that HMs are dynamically regulated during plant stress responses. Research has demonstrated that drought stress can alter histone acetylation, leading to upregulation of stress-responsive genes [26,79,80]. Their research findings demonstrate the significance of HMs in plant stress responses and offer insights into the underlying regulatory mechanisms. In plants, HM has been found to play a significant role in stress response when the stressors are biotic or abiotic. For instance, *trichostatin A*, which is a histone deacetylase inhibitor, has been demonstrated to enhance resistance to the fungal pathogen *Botrytis cinerea* in *Arabidopsis thaliana* [79,81]. 

Furthermore, abiotic stress factors like drought, salinity, extreme temperatures, and heavy metals trigger complex signaling pathways in plants, leading to changes in histone modifications. Studies have shown that drought stress increases histone H3 acetylation levels, affecting stress-responsive genes in *Arabidopsis thaliana* [78,82]. This acetylation is linked to the activation of stress-responsive genes, implying a direct relationship connecting histone changes and the plant’s ability to adapt to water deprivation. 

Similarly, salt stress has been found to induce changes in histone methylation patterns, affecting the expression of genes involved in ion homeostasis and osmotic regulation [83]. Cold stress increases chromatin accessibility in potato (*Solanum tuberosum*) via bivalent histone modifications (H3K4me3 and H3K27me3) of activated genes [84]. In fact, SHORT LIFE, a plant-specific methylation reader protein that identifies both active (H3K4me3) and repressive (H3K27me3) marks, was recently discovered [83]. Extreme temperatures modulate heat stress-responsive genes, highlighting the dynamic nature of histone modifications [22,78,85]. Histone acetylation is crucial in a plant’s stress response because studies have shown that HDA6 histone deacetylase controls stress-responsive genes and is critical for drought tolerance in *Arabidopsis thaliana* [45,80,86]. However, *histone methylation* (HMT) is considered a complex modification that can activate or repress gene expression [79], in which the degree of methylation and specific residue modification are vital factors [85]. On the other hand, histone phosphorylation through protein kinases is crucial for plant stress responses, like the H3S10ph phosphorylation induced by salt, cold, and drought stress, potentially leading to upregulation or silencing of stress-responsive genes [87]. The MAPK (mitogen-activated protein kinase) cascade is believed to play a role in histone phosphorylation and gene expression changes during stress responses [78,88]. HMs like acetylation, methylation, ubiquitination, sumoylation, and ADP-ribosylation can affect plants’ gene expression and chromatin structure. They play a role in stress-signaling pathways, although their exact function in plant stress response still needs to be fully understood [87].

#### Mechanisms of Histone Modification in Plant Stress Response

The precise mechanisms by which histone modifications regulate gene expression during plant stress responses are still being elucidated. However, several key mechanisms have been proposed based on studies of various plant species. One mechanism involves the recruitment of specific transcription factors or co-regulators to stress-responsive genes through the recognition of specific histone modifications with different environmental challenges (Table 2). To reduce agricultural losses, it is crucial to cultivate stress-tolerant crop varieties that can flourish in tough environments [89], and to accomplish this feat, it is essential to delve deeper into how plants react to stress and how chromatin states and histone modifications modulate gene expression [90,91]. For example, the binding of the WRKY transcription factor to H3K4me3 marks has been shown to activate the expression of stress-related genes in *Arabidopsis* [85]. Studies have revealed the significant role that post-translational modifications (PTMs) play, including seed formation, flowering, and responding to plant stresses [92]. Recent research found that reducing H3K27me3 deposition within the gene body region of drought stress-responsive TFs led to *Arabidopsis* drought stress tolerance [93]. Additionally, inoculation with *B. cinerea* was observed to significantly upregulate the genes DES, DOX1, and LoxD, which encode essential enzymes in the oxylipin pathway, as well as WRKY75, which encodes a stress-responsive TF in tomato *(Lycopersicum esculentus*) [94]. The activation of all pathogen-induced genes coincided with an increase in H3K4me3 and H3K9ac levels. In reaction with Pst DC3000, the same genes were activated. An elevation in H3K4me3 and H3K9ac was also found with this pathogen; however, it was substantially smaller than with *B. cinerea*-coupled DES and DOX1. WRKY75, on the other hand, exhibited a large rise in both histone marks along the gene.

Various abiotic factors, such as heat, salt, or limited water, can increase histone modification on a global scale, particularly in *Arabidopsis* with 12 different genetic families [103] (Table 3). Plants have eight histone lysine methylation sites: H3K4, H3K9, H3K26, H3K27, H3K36, H3K79, H4K20, and H1K26. Six arginine methylation sites are also present: H3R2, H3R8, H3R17, H3R26, H4R3, and H4R17 [13,83,104,105]. However, when *Arabidopsis* experiences drought stress, it results in an improvement in H3K4me3 and H3K9ac in the promoter areas of stress-responsive genes like RD20, RD29A, RAP2.4, and RD29B [106,107]. Furthermore, drought stress causes histone H3K4me1, H3K4me2, and H3K4me3 modifications throughout the *Arabidopsis* genome [108]. In rice seedlings under drought stress, 4837 genes exhibit differentially modified H3K4me3 [109,110]. Another mechanism involves the establishment of a “histone code” where multiple HMs act in conjunction to regulate gene expression. For instance, the interplay between H3K4me3 and H3K27me3 marks has been implicated in balancing gene activation and repression during stress responses. Additionally, histone modifications can also influence chromatin structure and accessibility by recruiting chromatin remodeling complexes or altering nucleosome stability.

The dynamic nature of plants enables them to adapt to environmental changes quickly. Although some progress has been made in understanding the impact of histone modifications on plant stress responses, many details remain to be clarified. In the future, researchers should concentrate on uncovering the exact mechanisms that govern histone modification-mediated gene regulation and identifying the specific readers and erasers of histone modifications that participate in stress signaling pathways.

### 2.3. Small RNA-Mediated Gene Silencing

Small RNA-mediated gene silencing and chromatin remodeling complexes are key players in plant stress mechanisms [124], like miRNAs and siRNAs (Figure 2), which regulate gene expression during stress [107]. These short, non-coding RNA molecules guide the RNA-induced silencing complex (RISC) to target mRNAs for degradation or translational repression. miRNAs are derived from endogenous hairpin-shaped precursors, while siRNAs are derived from exogenous double-stranded RNA or endogenous long double-stranded RNA precursors [125]. Both types of small RNAs function by base pairing with target mRNAs, resulting in mRNA cleavage or translational inhibition [106]. According to studies by Zhou et al. [126] and Singroha et al. [127], during drought stress, plants generate reactive oxygen species (ROS) in their chloroplasts and peroxisomes. However, to combat the damaging effects of ROS on cells, plants generate antioxidative enzymes such as superoxide dismutase, peroxidase, catalase, glutathione reductase, and ascorbate peroxidase [126,128]. Interestingly, some plant miRNAs, such as miR398 and miR528, are known to regulate oxidative stress networks [127,129,130]. 

Recent findings also indicate that stress-responsive miRNAs modulates plant stress tolerance by targeting stress-related genes [131]. Emerging evidence suggests that stress-induced lncRNAs regulate stress-responsive genes and pathways [131]. These RNAs can bind to messenger RNAs (mRNAs) and prevent their translation into proteins, or they can induce the degradation of specific mRNAs [132]. MiRNAs are used by plants to adapt to abiotic stress, such as drought and heat, and miR159 has been shown to modulate drought tolerance genes in *Arabidopsis thaliana* [33,87,132].

In plant stress response, small RNAs can be generated from stress-responsive genes or transposable elements that are activated under stress conditions [131]. Plants use small RNA molecules to regulate stress-responsive genes, where the mechanism enables them to adapt and thrive in challenging conditions. One example is miR398, which becomes active in oxidative stress and helps target the transcripts of copper superoxide dismutase (CSD) gene in *Arabidopsis thaliana* [127,132]. By repressing CSD expression, miR398 improves plant sensitivity to oxidative stress. 

Small RNAs regulate genes at both the transcriptional and post-transcriptional stages [107] and are also active in chromatin remodeling complexes, which alter chromatin structure to enable or restrict regulatory protein access to DNA, which, in turn, modifies gene expression [39,131]. In this RNA pathway, siRNAs produced by transposable elements or other repetitive sequences guide DNA methyltransferase DRM2 to specific genomic locations, resulting in gene silencing [133]. The Rd DM pathway has been related to plant stress response, regulating stress-responsive genes such as those producing heat shock proteins in *Arabidopsis* [116]. Furthermore, small RNAs can also interact with other chromatin remodeling complexes, such as histone modifiers, to regulate gene expression. Small RNAs can direct histone modifiers to specific genomic areas, causing HMs and gene expression to vary [106]. In *Arabidopsis*, for example, miR156 controls flowering time by targeting Squamosa promoter-binding protein-like transcription factors [107]. The interaction between miR156 and SPLs influences HMs at the flowering locus, thereby modulating flowering time. 

In conclusion, the stress response of plants heavily relies on the critical role played by small RNA-mediated gene silencing and chromatin remodeling complexes. Small RNAs effectively regulate gene expression by targeting mRNAs by directing and guiding complexes that modify chromatin structure to specific locations within the genome. Such mechanisms allow plants to adapt to varying environmental conditions by precisely adjusting their stress responses.

### 2.4. Chromatin Remodeling Complexes

In plants, chromatin remodeling complexes play an important role in the epigenetic control of gene expression, particularly in response to stress [37,72,134]. These complexes are in charge of modifying chromatin shape, impacting DNA accessibility to transcriptional machinery and regulatory proteins [134]. In the context of plant stress, such as abiotic and biotic challenges, chromatin remodeling complexes play a role in modifying the expression of stress-responsive genes, affecting the plant’s capacity to adapt and survive under adverse conditions [135]. Plants respond to stress by reprogramming gene expression patterns through chromatin remodeling complexes, which promote structural modifications to activate or repress stress-responsive genes, ensuring survival and fine-tuning gene expression [87].

Chromatin remodeling complexes in plant stress responses alter gene expression by using ATP hydrolysis energy to slide, evict, or change nucleosome composition [128]. Histone-modifying enzymes catalyze posttranslational alterations of histone proteins, stimulating or inhibiting gene transcription. When extremely condensed, the chromatin arrangement prohibits transcription factors, polymerases, and other nuclear proteins from accessing DNA because some chromatin structural changes occur as a result of stress signals, allowing DNA to become accessible [16]. Chromatin remodeling complexes play a crucial role in epigenetic regulation, enabling fast, reversible changes in gene expression in response to plant stress [136]. This dynamic control allows plants to effectively respond to various stressors without altering the underlying genetic code. To organize complete responses to stress, chromatin remodeling complexes collaborate with other epigenetic processes, including DM and small RNA-mediated silencing pathways. Cross-talk between these multiple levels of epigenetic regulation leads to plant stress response, resilience, and adaptability. Furthermore, new data reveal that environmental signals can impact the activity and specificity of chromatin remodeling complexes, establishing a direct relationship between external stimuli and epigenetic changes that influence stress-responsive gene expression [119,137].

## 3. Recent Discoveries of Plant Epigenetic Regulation in Response to Plant Stress

Plant epigenetic regulation is a rapidly developing field that has garnered significant attention in recent years due to its potential to improve crop yields, disease resistance, and environmental adaptability (Table 4). In response to epigenetic regulation under stress, we will highlight recent studies published within the last 5–10 years that have significantly contributed to our understanding of plant epigenetic regulation under stress. Based on reports from [34,78,138,139], DM and histone acetylation are utilized to control chromatin structure, which can cause long-term alterations in DNA structure, which can either activate or suppress the expression of genes. DNA methyltransferases target two types of cytosines: CpNpG and CpG. Environmental changes can cause cytosines to alter gene expression without changing the DNA sequence [67].

According to Zhang et al. [109], using mature embryos as a starting point is common when attempting to form a callus in rice (*Oryza sativa* L.). However, TSA treatment appears to inhibit the formation of this callus. According to research by the same authors, OsHDA710 plays a crucial role in reducing the acetylation levels of OsARF18 and OsARF22. Interestingly, research [144] has shown that modest dosages of TSA can induce callus and shoot development in mature wheat embryos. In contrast, excessive dosages of TSA can impede these activities. Furthermore, sodium butyrate, a histone deacetylase inhibitor, has been shown to improve wheat (*Triticum aestivum* L.) regeneration [144]. Due to the versatile nature of histone acetylation modifiers, HDACs have varied roles in regenerating different explants in different species by modifying various lysine residues of different histones [18].

Another study conducted by Zhang et al. [44] who investigated DM mechanisms in interceding the epigenetic reaction to drought stress in *Arabidopsis thaliana*. The authors found that drought stress led to widespread changes in DM patterns, particularly in regions consociated with transposable elements and gene bodies. They observed changes in DM and gene expression under stress conditions. Recent studies confirmed that abiotic stresses can cause genes in plants to become more methylated, leading to a decrease in genome activity. On the other hand, for plants cultivated under optimal situations without stress, methylation levels are lower, and gene expression is better. The presence of DM is required for embryonic development, X inactivation, genomic imprinting, proliferation, and cellular differentiation [67,145]. Methylation sites are often found in gene promoters, and they are targeted by genomic methylation situated in condensed chromatin regions rich in transposons and repetitive elements [36].

Another important study was conducted by Akhter et al. [5]; Liu et al. [18]; and Pathak et al. [74], who examined the significance of various stressors on the epigenetic landscape of rice (*Oryza sativa*). The authors utilized ChIP-seq (Chromatin immunoprecipitation sequencing) and reduced representation bisulfite sequencing to profile DM and histone modifications in rice plants experiencing drought salinity, and high-temperature stress [5]. They found that each type of stress-induced distinct epigenetic change, including differentially methylated regions (DMRs) and histone modifications, was often associated with specific genes required in stress response pathways. In addition, Li et al. [27] and Hu et al. [79], investigated histone acetylation mediating epigenetic reactions to drought stress in *Zea mays*, and found that drought stress led to increased histone acetylation at specific genomic regions, which in turn activated the expression of stress-responsive genes involved in water conservation and stress tolerance. They also observed that histone deacetylase inhibitors could enhance drought tolerance in maize plants, suggesting that targeting histone acetylation may be a promising approach for improving crop stress tolerance.

In addition to these studies, several other recent works have also made significant impacts on our knowledge about plant epigenetic regulation under stress. For example, Ferdous et al. [132] and Statello et al. [146] investigated the effects of non-coding RNAs in regulating epigenetic marks on gene regulation under drought stress in *Arabidopsis*, while Ashapkin et al. [29] explored the interaction between histone modification and DM in regulating gene expression under abiotic stress in rice. Overall, this recent research has advanced our knowledge about the epigenetic regulation of plants under stress, revealing complex epigenetic mechanisms that play critical functions in mediating the reaction to environmental cues. By elucidating the underlying epigenetic regulatory networks, these studies have shown related knowledge of novel strategies for improving crop stress tolerance and environmental adaptation.

### Breakthrough Findings in Epigenetics and Plant Stress Responses

Plant stress responses involve complex physiological and molecular differences that enable plants to survive and adapt to adverse environmental conditions. Recent breakthrough findings in the field of epigenetics have shed light on the critical role of epigenetic regulation in plant stress responses. Based on this review, here are some of the key discoveries and breakthroughs in epigenetics in relation to plant stress responses: 

Epigenetic regulation of stress-related genes: Drought stress triggers changes in DM patterns in stress-related genes of plants, which increases their expression and resilience to drought stress [147,148,149]. Similarly, other types of stress, such as heat stress and pathogen attack, also induce epigenetic changes in stress-related genes [150]. Barley unequivocally displayed elevated DM levels in the promoter region of HvCKX 2.1 when subjected to drought stress, according to [42]. These resulted in a quick induction of shoots due to heightened cytokinin levels [42]. Additionally, the drought-induced mutation was heritable in rice, with the altered DM pattern persisting in future generations [151]. Rice genes modified with DM show altered transcription levels post-Cd treatment, linked to transcriptional differences in stress-responsive genes involved in metal transport, metabolic processes, and regulation [75]. 

Epigenetic memory: Another important finding is the concept of “epigenetic memory”, which refers to the idea that plants can retain an epigenetic imprint of past stress experiences and use it to fine-tune their stress responses in the future. This phenomenon has been observed in Arabidopsis thaliana, where exposure to drought stress leads to long-lasting modifications in DM patterns at the enhanced places of stress-related genes, even after the stress has been removed [45,86].

Epigenetic regulation of hormone signaling: Hormones play a critical role in plant stress responses, and recent research has shown that epigenetic regulation of hormone signaling is involved in plant stress adaptation. For example, studies have found that DM and HM regulate abscisic acid (ABA) signaling and that these epigenetic changes play a crucial role in ABA-mediated stress responses [152,153].

Epigenetic regulation of secondary metabolism: Plants have developed a sophisticated defense mechanism that utilizes secondary metabolites, such as phenolic compounds and alkaloids, against potential threats from pathogens and herbivores. Emerging research suggests epigenetic regulation also plays a crucial role in producing these compounds [154,155,156]. Specifically, studies demonstrate that DM and histone modifications improve the gene modification that produces anthocyanins, a particular phenolic compound that safeguards plants from UV radiation and oxidative stress [44,154].

Epigenetic regulation of stem cell maintenance: Stem cells are essential for the germination of plants and stress response, and recent research has shown that epigenetic regulation is included in stem cell maintenance. For example, studies have found that DM and HM are involved in the influence of stem cell fate decisions in *Arabidopsis thaliana* [12,25,156].

## 4. Implications and Applications of Plant Epigenetic Regulation

Mechanisms of plant epigenetic regulation in response to stress have significant implications for agriculture and crop improvement. By deciphering the epigenetic regulatory networks involved in stress responses, researchers can potentially develop strategies to enhance stress tolerance in crops. For instance, manipulating DM patterns or histone modifications could be used to improve crop resilience to drought, salinity, or pathogen attacks. Furthermore, identifying stress-responsive ncRNAs and their targets could provide new avenues for genetic engineering and breeding programs aimed at enhancing stress tolerance in crops [37]. Recent advancements in the development of engineered DNA-binding domains have made it possible to achieve even greater precision in locus-specific epigenetic breeding technology [157]. However, plants have the unique advantage of not requiring germline maintenance while allowing for transmission of epigenetic information during gametogenesis, although this transmission can be unstable in plants [124,136,158,159]. 

**Figure 3:**
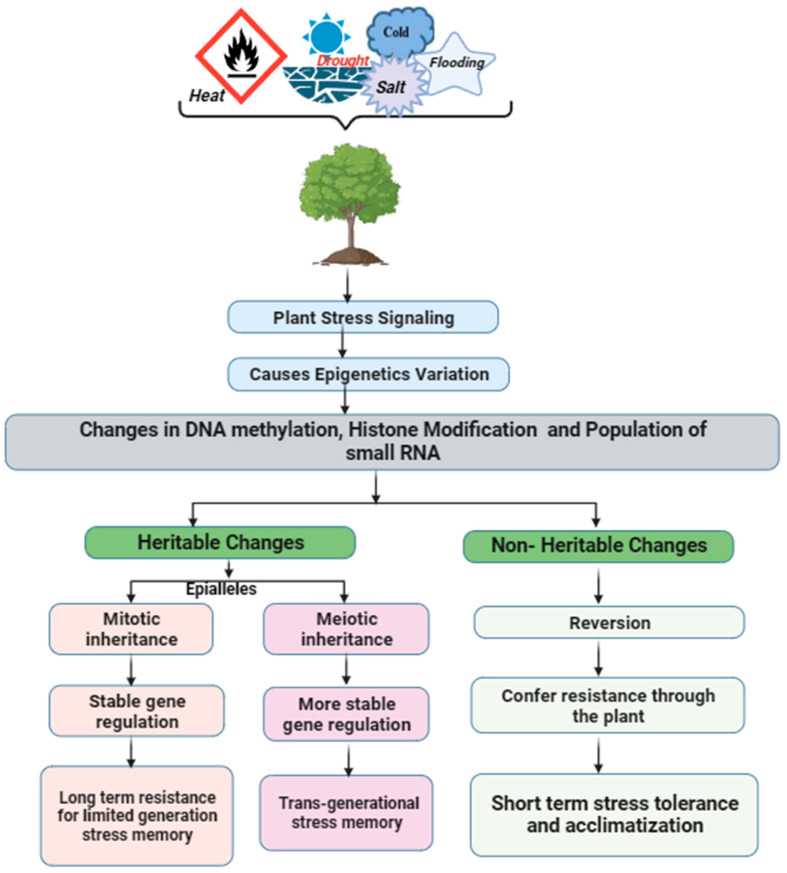
Responses of Epigenetic Variation to Stress Tolerance in Plants.

Furthermore, epigenetic modifications can also be inherited across generations, providing a mechanism to enhance the transgenerational stress tolerance of plants [157]. Epigenetic inheritance transfers modifications across generations without DNA sequence changes [160], and it aids stress adaptation in different plant species. According to Schmid et al. [161], Herrera et al. [162], and Springer and Schmitz [163], plants can decide what information to keep or discard, and when exposed to stress, plants can create epigenetic stress memories, also called epialleles, which can be temporary or permanent [14,156]. These memories carry on beyond the initial stressor and can even be passed down to future generations, facilitating plant adaptability and evolution [5,164]. It is worth noting that any fleeting memories revert once the stress is no longer present (Figure 3). This is feasible because plants acquire their germ lines late in development and recall the problems they face throughout their lives, which are passed on to offspring via epigenetic processes [165]. By understanding the mechanisms underlying transgenerational epigenetic inheritance, researchers can potentially develop breeding strategies that exploit this phenomenon to enhance stress tolerance in crops [37,166,167].

While most studies on epigenetic mechanisms have been conducted in *Arabidopsis*, researchers are increasingly interested in investigating epigenetics in other plants to breed climate-resistant crops and adapt to changing climates [124,148,168]. Climate stress conditions can severely impact crop yield and quality, leading to significant economic losses [156]. Genetic engineers can introduce genes encoding enzymes involved in epigenetic modifications into crop plants. For example, the overexpression of genes encoding DNA methyltransferases, enzymes responsible for adding methyl groups to DNA, has been demonstrated to improve stress tolerance in various plant species [53,169]. Increased DM levels have been associated with improved drought tolerance in crops such as rice and maize [14]. In addition to genetic engineering, epigenetic regulation mechanisms can also be exploited through traditional breeding strategies [170]. Breeding for stress tolerance involves selecting and crossing plants with desirable traits and then selecting offspring with improved stress tolerance [12]. 

Additionally, researchers have found that epigenetic-related traits can be improved through artificial selection, leading to their inclusion in crop breeding processes where scientists can improve energy use efficiency in canola by selecting plants with the highest and lowest cellular respiration in an isogenic doubled haploid line [37,171]. Knowledge of epigenetic regulation mechanisms can help breeders identify and select plants with favorable epigenetic modifications associated with stress tolerance [169]. For example, breeders can screen for plants with increased DM levels or altered chromatin structure in response to stress [136]. These plants can then be used as parents in breeding programs to develop stress-tolerant crop varieties.

DM affects gene expression by inhibiting the binding of transcription factors, making it harder for genes to function [138]. However, researchers have further investigated stress-responsive genes and their associated DM patterns in stress-tolerant plant varieties [23,56,63]. These have helped in identifying specific epigenetic modifications associated with stress tolerance and enabling the targeted manipulation of these modifications in crops through genetic engineering [44,63]. Similarly, histone modifications are proteins around which DNA is wrapped. Modifying these proteins can affect how DNA is packaged and accessed by the cellular machinery [26]; by understanding and manipulating HM, researchers can potentially enhance stress tolerance in crops. Plant stress tolerance can be enhanced in crops by increasing the expression of enzymes involved in histone acetylation and methylation modifications, which have been linked to stress tolerance [27,167,172].

Additionally, DNA binding domains can actuate or trigger specific chromatin marks at specific locations in a plant’s genome [165]. As a result, this can influence the plant’s phenotype as it develops and encounters stressors [38,157]. Scientists have utilized epigenetic single nucleotide polymorphisms to alter particular genes and utilize epigenetic variations to create new transgene-free breeding methods for crops [17,136]. Additionally, small RNAs can bind to target mRNA molecules and either promote their degradation or suppress translation, thereby regulating gene expression and enhancing stress tolerance [131]. In the near future, it will be critical for researchers to concentrate on epigenetic modifications and their impact on aiding plants in adapting to climatic changes, which could ultimately create new stress-resistant crops. Nonetheless, further studies are crucial to regulate epigenetics in plants for both functional research and crop enhancement.

Conclusively, information on epigenetic regulation principles ensures stress-tolerant crops through genetic engineering or breeding strategies. Genetic engineering allows for the direct manipulation of genes involved in epigenetic modifications, while breeding strategies can exploit natural variation in epigenetic modifications associated with stress tolerance. By understanding and manipulating DM patterns, HM, and small regulatory RNAs, researchers can develop genetically engineered or selectively bred stress-tolerant crop varieties. These could have significant implications for food security and the sustainability of agriculture and environmental challenges.

### 4.1. Potential Implications of Plant Epigenetic Regulation in Response to Stress

Understanding plant epigenetic regulation in response to stress is greatly important for environmental sustainability, agriculture, and crops. Some of the potential implications gathered by this review are itemized below:

**Enhanced Stress Tolerance**: By unraveling this, researchers can identify key genes and pathways that can be targeted for crop improvement. Traditional breeding methods have limitations in terms of the speed and precision with which desired traits can be introduced into crops. Epigenetic modifications offer an additional layer of control over gene expression, allowing for more targeted manipulation of stress-responsive genes [173]. By identifying and modifying key epigenetic marks associated with stress tolerance, researchers can enhance the ability of crops to withstand adverse conditions. These could lead to the advancement of resilient plant species that are equipped to cope with stress-induced varieties and ultimately ensure food security. 

**Sustainable Agriculture**: Epigenetic regulation provides a potential avenue for sustainable agriculture practices. By studying how plants respond to different stressors at the epigenetic level, scientists can identify specific molecular pathways and regulatory networks involved in stress adaptation [173]. This knowledge can then be used to design management strategies that minimize adverse stress impacts on crop quality and yield [74]. For example, by manipulating epigenetic marks associated with water use efficiency, farmers can reduce irrigation requirements without compromising crop productivity. These would not only conserve water resources but also reduce energy inputs and mitigate environmental pollution associated with excessive irrigation [62]. 

**Precision Agriculture**: Epigenetic insights can contribute to the development of precision agriculture techniques. By understanding the specific genes and pathways involved in stress responses, farmers can make informed decisions about crop management practices [12]. These include optimizing irrigation schedules, applying targeted treatments, and implementing site-specific strategies to maximize crop performance while minimizing resource use.

**Climate Change Adaptation:** Agriculture faces significant hurdles due to climate change’s impact, which includes extreme weather events that are more frequent and severe [156]. However, a more profound comprehension of plant epigenetic regulation can help pinpoint genetic traits and molecular mechanisms that allow plants to adapt to changing environmental circumstances. Such insights can direct breeding programs toward creating plants with stress tolerance that can thrives in future climatic scenarios [12,20,157].

**Conservation and Biodiversity**: The knowledge about plant conservation and biodiversity preservation can be applied to ecosystem restoration efforts, such as the reintroduction of native plant species or the rehabilitation of degraded lands [27,169]. Understanding how epigenetic modifications influence plant interactions with other organisms, such as pollinators or beneficial soil microbes, can also inform conservation strategies aimed at preserving biodiversity and promoting ecological resilience. 

In summary, understanding plant epigenetic regulation in response to stresses contributes to the production of plant stress-tolerance species, optimizes agricultural practices, and aids in ecosystem restoration efforts. By harnessing the power of epigenetics, scientists can pave the way for a more sustainable and resilient agricultural system that can adapt to the challenges posed by climate change and other environmental pressures [156]. By manipulating genes encoding chromatin remodeling proteins, researchers can potentially enhance stress tolerance in crops [170]. Understanding these mechanisms not only advances our knowledge of plant biology but also holds great potential for improving crop resilience to environmental stresses.

### 4.2. Limitations/Challenges in Translating Research Findings into Practical Applications

Translating research findings on epigenetic regulation into practical applications for developing stress-tolerant crops faces a few limitations and challenges. These challenges arise from the complexity of the epigenetic regulation mechanisms and the need for extensive testing and validation before widespread adoption. We discuss some of the challenges/limitations as compiled in this review below:

**Complexity of Epigenetic Regulation:** Epigenetic regulation is a complex process involving DM, histone modifications, and non-coding RNAs. However, the interaction and influence of these markers on gene expression still need to be fully understood. Moreover, significant tissue, developmental, and environmental variations add to the complexity of interpreting epigenetic data [40]. The application of genetic engineering in agriculture requires regulatory approval and adherence to safety protocols. Translating research findings into practical applications involves extensive testing to determine the safety and environmental impacts of genetically modified crops [12,25,31,80]. Regulatory frameworks and public acceptance play significant roles in determining the adoption of genetically engineered crops. Moreover, understanding the specific mechanisms underlying stress tolerance in crops requires adequate knowledge of the intricate interactions between epigenetic and genetic factors. This complexity poses a significant challenge when translating research findings into practical applications for crop improvement.

**Lack of Standardized Epigenomic Techniques:** Another limitation in translating epigenetic research into practical applications is the need for standardized techniques for studying the epigenome. Different methodologies can yield varying results, making it difficult to compare data across studies or establish consistent guidelines for crop breeding programs [174,175]. However, these techniques often require specialized equipment, expertise, and substantial resources. Standardizing protocols and developing cost-effective methods for large-scale epigenomic analyses would facilitate the translation of research findings into practical applications for crop improvement. There is still much to learn about the relationship between specific epigenetic modifications and stress tolerance [176]. More research is needed to fully comprehend the epigenetic landscape and its influence on crop responses to different stresses.

**Epigenetic Plasticity and Transgenerational Effects:** Epigenetic modifications can exhibit plasticity, meaning they can be reversible and responsive to environmental cues. This plasticity allows plants to adapt to changing conditions, including stress. However, it also presents challenges when trying to develop stress-tolerant crops through epigenetic regulation [177]. The plasticity of the epigenome means that the same stressor can induce different epigenetic changes in different individuals or populations. This variability makes it challenging to identify consistent epigenetic markers associated with stress tolerance [170]. Additionally, the transgenerational effects of epigenetic modifications further complicate the translation of research findings into practical applications.

**Transferability across different crop varieties:** Epigenetic modifications can be inherited across generations, potentially influencing the phenotype of the offspring [160]. However, the stability and heritability of these transgenerational effects still need to be better understood. Understanding the dynamics and stability of transgenerational epigenetic effects is crucial for developing reliable strategies for crop improvement. Research findings on epigenetic regulation mechanisms often focus on a limited number of plant species or varieties. Implementing these findings into practical applications would require successfully transferring the knowledge across different crop varieties. Each crop has its own unique genetic and epigenetic makeup, making it challenging to apply universal solutions across diverse plant species. 

**Understanding complex epigenetic interactions:** Epigenetic regulation is a complex process involving various modifications and interactions. Deciphering the intricate mechanisms and identifying specific genomic regions that affect stress tolerance requires extensive research and experimentation. The complexity of these interactions makes it challenging to predict the outcomes of genetic modifications accurately.

**Complex genetic and environmental interactions:** Crop responses to stress are influenced by various genetic and environmental factors. Genetic engineering or breeding strategies aimed at improving stress tolerance must consider the interactions between multiple genes, their epigenetic regulation, and the complex environmental conditions encountered in different regions or seasons [167].

In summary, translating epigenetic regulation research findings into practical applications for developing stress-tolerant crops faces several limitations and challenges. The complexity of epigenetic regulation, the lack of standardized techniques, and the plasticity and transgenerational effects of epigenetic modifications all contribute to these challenges. Addressing these limitations and challenges requires continued research efforts, technological advancements, collaboration between scientists and breeders, and effective regulatory frameworks. Additionally, interdisciplinary approaches that combine genomics, epigenomics, bioinformatics, and plant physiology will be crucial for optimizing the translation of epigenetic research into practical applications for developing stress-tolerant crops.

## 5. Conclusions

Plant epigenetic regulation in response to stress is a significant area of research with great potential for future applications in agriculture. Epigenetic modifications play a key role in modulating gene expression and can influence plant responses to various stressors, including drought, heat, salinity, and disease. Understanding the epigenetic mechanisms underlying stress tolerance in plants can offer insights into enhancing crop resilience and improving agricultural productivity. This review confirmed that one significant aspect of plant epigenetic regulation in stress response is the potential for transgenerational inheritance of stress memory. This indicates that epigenetic marks can be heritable, meaning stress experiences in one generation can influence the stress tolerance of subsequent generations. This transgenerational memory enables plants to better adapt to recurring stress conditions and may provide a mechanism for the rapid adaptation of crops to changing environments. We found that plant epigenetics regulate stress responses by modifying gene expression without altering DNA sequence. Rather than relying solely on genetic changes, which occur over longer evolutionary timescales, plants can dynamically adjust their gene expression patterns through epigenetic modifications to cope with stress conditions. Additionally, epigenetic plasticity offers a more rapid and flexible response to changing environments, providing a survival advantage for plants. 

The potential applications of understanding plant epigenetic regulation in stress response are wide-ranging. Researchers can apply this knowledge to develop stress-tolerant crop varieties through genetic engineering or breeding strategies. By modulating the epigenome of crops, researchers aim to enhance stress tolerance, improve crop yields, and increase the resistance of plants to biotic and abiotic stresses. These have the potential to improve global food security by enabling agricultural systems to cope with increasingly challenging environmental conditions. Furthermore, this review further confirmed that the field of epigenetics opens up avenues for targeted and precise gene regulation, allowing for the development of crop varieties with specific responses to stress. For example, researchers can use epigenetic modifications to fine-tune the expression of stress-responsive genes or alter the sensitivity of plants to environmental signals. This level of precision can be harnessed to optimize crop performance and resilience to stress conditions. Finally, plant epigenetic regulation in response to stress holds immense significance in agriculture, and understanding its mechanisms in stress tolerance can revolutionize crop breeding and genetic engineering strategies, leading to the evolution of stress-tolerant crops and ensuring sustainable food production in the face of climate change and other environmental challenges. Future research in this field will continue to unveil the intricacies of epigenetic regulation and its potential applications in crop improvement.

## Figures and Tables

**Table 1:** Studies on DNA methylation in different plant species under stress.

Stress	Plants	Processes	Mechanisms/Responses	References
Heat	*Zea mays*	DM analysis	Enhancing adequate tolerance to heat and increase in methylation	[51]
	*Brassica napus*	Msap	Both the heat-tolerant genotype and heat-sensitive genotype improve the DM	[52]
	*Gossypium*	Regulation of anther development	Increase in DM	[47,53]
	*Arabidopsis thaliana*	Mouse-ear cress (A. thaliana)	The process of increasing the activity of epigenetic modulators.	[54]
Drought	*Medicago sativa*	DM change	Decrease in DM processes	[55]
	*Oryza sativa*	Msap	Genome site-specific methylation deference	[56,57]
	Physcomitrella patens and *Arabidopsis thaliana*	DM of gene promoters	Enhanced ABA represses gene expression	[42,58]
	*Arabidopsis thaliana*	Drought transcriptome analysis	Improved water retention, increase transposon expression and limit global genome methylation	[29,43]
	*Zea mays*	Transcriptome, miRNA, DM analysis	Promote water retention	[14,59,60]
	*Populus trichocarpa*	BS-seq	Enhanced the methylated cytosines amount	[61]
Heavy metals	*Arabidopsis thaliana*	Msap	Increase in DM	[34]
	*Groceria Dura*	Msap	Enhanced the DM	[34,62]
	*Trifolium repens*	DM Analysis	Hypomethylation in tolerant upon prolong exposure	[63]
	*Oryza sativa*	Msap	DM	[64]
Cold	*Prunus simonii*	Msap	Cytosine methylation	[57]
	*Alpine*	Msap	Cytosine methylation	[65]
Salt	*Glycine max*	Expression of various transcription factors	Demethylation and hypomethylation tolerant and susceptibility	[66]
	*Oryza sativa*	ELISA-based assay	Hypomethylation intolerant cultivar	[63]
	*Brassica napus*	Msap	Hypomethylation intolerant and hypermethylation in sensitive cultivars	[67]

Keys: Msap = Methylation sensitive amplification polymorphism; DM = DNA methylation.

**Table 2:** Histone modification responses to stress in plant species.

Stress Response	Plant Species	HM Mechanisms	References
Drought	*Gossypium hirsutum*	Improved drought tolerance by decreasing H3K9ac levels in the GhWRKY33 promoter via GhHDT4D, an HD2 histone deacetylase.	[95]
	*Triticulum aestivum*	Drought stress downregulated 5 HDA genes and upregulated TaHAC2 in drought-resistant BL207	[96]
	*Dendrobium hirsutum*	Under drought stress, the DoHDA10 and DoHDT4 genes are expressed in the roots, stems, and leaves.	[97]
	*Arabidopsis thaliana*	HDA9 reduces plant drought sensitivity via H3K9ac in 14 genes during water deficit	[98]
	*Brassica rapa*	Drought treatment significantly increases the expression of 9 HAT genes, aiding drought stress response and adaptation.	[99]
	*Oryza sativa*	Nine HAT genes are triggered under drought conditions, some with MBS drought-sensitive elements in their promoter regions	[100]
Heat	*Arabidopsis thaliana*	HDA9 removes the histone variant H2A.Z from the YUC8 nucleosome, activating transcription via phytochrome interacting factor4 and mediating thermo-morphogenesis	[101]
		HDA9 interacts with PWR and regulates thermomorphogenesis via phytochrome interacting factor4 and YUC8 genes.	[102]

**Table 3:** Enzymatic groups catalyzing Histone modifications in Arabidopsis.

Histone Group	Gene	Target	Role in Stress	References
Deacetylases	At3G44680	H3K9	Improve salinity and drought resistance	[98,111]
	At3G44750	H3K18	Repressed in the activation of ABA pathways and salt tolerance	[30]
	At2G27840	H3K27	Drought and cold resistance and salinity tolerance	[112]
	At3G18520		Drought resistance	[30,113]
	At5G09230	H3K9	Ethylene response	[44]
	At5G63110	H3K9,	Pathogen defense, heat and cold tolerance	[114]
Lysine Methyltransferase	At5G42400	H3K4	Enhanced plant immunity and heat defense	[13,115]
	At4G27910	H3K4	Drought resistance	[116]
	At2G31650	H3K4	Enhance the tolerance of heat, osmotic reactions, and dehydration of plant stress	[13]
	At4G31120	H4R3	Salinity tolerance and drought resistance	[117]
	At1G77300	H3K36	Immunity defense	[115]
	At5G53430	H3K4	Drought resistance	[116]
Acetyltransferases	At3G12980	H3K9	Ethylene response	[118]
	At1G79000	H4K14, H3K9	Heat tolerance and ethylene response	[118,119]
	At5G50320	H3K56	Efficient UVB light responses	[120]
	At5G09740	H4K5	Adequate UV light responses, repair of DNA	[121]
	At3G54610	H3K14	Salt tolerance, cold tolerance, and decreasing heat stress	[108,122]
	At5G64610	H4K5	UV light responses, repair of DNA	[121]
Demethylases	At4G00990	H3K9me2	Activation of the ABA pathways and drought tolerance	[104]
	At4G20400	H3K4me1/2/3	High temperature and decreasing the salt stress	[123]
	At1G63490	H3K4me1/2/3	Dehydration	[110]
	At2G34880	H3K4me1/2/3	High temperature and salinity tolerance	[105,123]
	At3G45880	H3K27me3	Cold tolerance and heat stress reduction	[122]

**Table 4:** Epigenetic Regulation Processes and Responses to different Plant Stress.

Plant Species	Epigenetic Process	Mechanism	References
*Zea mays*	Histone modification	The H2A variant plate_number_1 exhibits differential expression in hybrid genotypes, affecting early seed germination.	[140]
	Small RNA	Mutation of the mop1 gene globally reduces 24 nt siRNA, allowing advantageous plant traits to persist through sustained gene expression and hybrid vigor	[60]
*Arabidopsis thaliana*	Small RNA	A correlation exists between DM, gene expression changes, and reduced 24 nt siRNA levels, which collectively enhance plant vigor.	[124]
	DM	Altered DM patterns, specifically mCG and mCHH islands, are linked to decrease the levels of RNA and increased biomass and seed yield heterosis	[141]
		The pathway known as RNA-directed DM(RdDM) is responsible for increasing the presence of DM in specific genes, promoting growth vigor in hybrids	[107]
	Histone modification	Flowering locus expression, regulated by H3K27me3 levels, delays flowering and enhances heterosis	[110]
		Reducing H3K9ac and H3K4me2 marks suppresses genes related to circadian clock and late elongated hypocotyl, while enhancing gene expression in chlorophyll biosynthesis and starch metabolism, promoting growth vigor.	[120]
*Oryza sativa*	Histone modification	Hybrid vigor shows a strong positive correlation with the H3K4me3 mark, which impacts gene expression, whereas it displays only minimal correlation with the H3K27me3 mark, contributing to growth vigor.	[74]
	Histone modification	Allele-specific histone modifications, like H3K36me3, regulate the expression of histone modifications in F1 hybrids, where epialleles play a significant role.	[109]
	DM	This process causes epigenetic changes that promote heterosis in genetically identical chromosomes across generations	[47]
*Brassica rapa* L. spp. *pekinensis*	Small RNA	Reducing miRNA cluster expression levels enhances photosynthesis and biomass.	[142]
*Brassica napus*	Small RNA	Heterosis in flower development is achieved by increasing small interfering RNA expression in hybrids and reducing transposable element expression through methylation changes.	[143]
